# Opposite roles of transcription elongation factors Spt4/5 and Elf1 in RNA polymerase II transcription through B-form versus non-B DNA structures

**DOI:** 10.1093/nar/gkab240

**Published:** 2021-04-20

**Authors:** Jun Xu, Jenny Chong, Dong Wang

**Affiliations:** Division of Pharmaceutical Sciences, Skaggs School of Pharmacy and Pharmaceutical Sciences, University of California, San Diego, La Jolla, CA 92093, USA; Division of Pharmaceutical Sciences, Skaggs School of Pharmacy and Pharmaceutical Sciences, University of California, San Diego, La Jolla, CA 92093, USA; Division of Pharmaceutical Sciences, Skaggs School of Pharmacy and Pharmaceutical Sciences, University of California, San Diego, La Jolla, CA 92093, USA; Department of Cellular and Molecular Medicine, University of California, San Diego, La Jolla, CA 92093, USA; Department of Chemistry and Biochemistry, University of California, San Diego, La Jolla, CA 92093, USA

## Abstract

Transcription elongation can be affected by numerous types of obstacles, such as nucleosome, pausing sequences, DNA lesions and non-B-form DNA structures. Spt4/5 and Elf1 are conserved transcription elongation factors that promote RNA polymerase II (Pol II) bypass of nucleosome and pausing sequences. Importantly, genetic studies have shown that Spt4/5 plays essential roles in the transcription of expanded nucleotide repeat genes associated with inherited neurological diseases. Here, we investigate the function of Spt4/5 and Elf1 in the transcription elongation of CTG•CAG repeat using an *in vitro* reconstituted yeast transcription system. We found that Spt4/5 helps Pol II transcribe through the CTG•CAG tract duplex DNA, which is in good agreement with its canonical roles in stimulating transcription elongation. In sharp contrast, surprisingly, we revealed that Spt4/5 greatly inhibits Pol II transcriptional bypass of CTG and CAG slip-out structures. Furthermore, we demonstrated that transcription elongation factor Elf1 individually and cooperatively with Spt4/5 inhibits Pol II bypass of the slip-out structures. This study uncovers the important functional interplays between template DNA structures and the function of transcription elongation factors. This study also expands our understanding of the functions of Spt4/5 and Elf1 in transcriptional processing of trinucleotide repeat DNA.

## INTRODUCTION

Transcription elongation is a dynamic process that is frequently interrupted by transcription obstacles, which may lead to transient transcription pausing or prolonged stalling (1). In eukaryotes, these obstacles include nucleosomes, DNA epigenetic modifications, DNA lesions, pausing sequences and non-B-form DNA structures (2–4). In particular, Holliday junctions (5), R-loops (6), H-DNA (7) and slip-outs (8) have been shown to block transcription elongation. Importantly, if left unresolved, these non-B-form DNA-induced transcription stalling can lead to transcription–transcription and transcription–replication collisions, which can result in genome instability and cellular dysfunction (4,9).

Spt5 is a conserved transcription elongation factor among all three domains of life (10). In eukaryotes, Spt5 and Spt4 form a complex (DSIF in human) with transcribing RNA polymerase II (Pol II) to regulate transcription elongation (11). Biochemical studies identified that Spt4/5 stimulates Pol II bypass of nucleosomal barriers (12,13) and pausing sequences (14) by suppressing either Pol II pausing or arrest. Importantly, previous genetic investigations revealed that Spt4/5 plays critical roles in the transcription of genes with repeated DNA (15–17).

Elf1, another conserved Pol II elongation factor, displays synthetic lethality with Spt4/5 in yeast (18). Elf1 works together with Spt4/5 to facilitate Pol II bypass of nucleosomal barriers, ensuring high transcription processivity on chromatin template (12). Importantly, Elf1 interacts with Pol II and Spt4/5 to stabilize Pol II elongation complex (19). In the Spt4/5–Elf1–Pol II structures, Elf1 is located between the Rpb2 lobe domain and the Rpb1 clamp-head domain of Pol II, bridging the Pol II central cleft. Thus, Spt5–NGN–KOW1, Spt4, Rpb2 protrusion and wall establish a ‘DNA exit tunnel (upstream DNA)’, whereas Elf1, Rpb2 lobe and Rpb1 clamp head compose a ‘DNA entry tunnel (downstream DNA)’ (19).

The instability of CTG•CAG trinucleotide repeat (TNR) sequences is associated with multiple neurological diseases such as Huntington disease (HD), myotonic dystrophy type 1 (DM1) and several forms of spinocerebellar ataxia (SCA) (20). The CTG•CAG trinucleotide repeat can form at least two different conformations during transcription: the B-form duplex tract and the non-B-form slip-out (20–22), which can be generated by separating the DNA duplex during transcription (20). Importantly, both the B-form duplex tract (23) and the non-B-form slip-out have been shown to impede Pol II transcription elongation with the transcription machinery from nuclear extracts (8,24).

Previous single-molecule and biochemical studies showed that Spt4/5 positively regulates transcription elongation of B-form DNA template by increasing the processivity of Pol II (25). However, there are no direct experimental data revealing the roles of Spt4/5 and Elf1 in Pol II transcription of both forms of CTG•CAG tri-nucleotide repeat DNA template. Here, we took advantage of an *in vitro* reconstituted system with purified proteins from budding yeast to investigate the functional role of Spt4/5 and Elf1 in Pol II transcription of CTG•CAG repeat. We designed two scenarios: first, we tested the effect of Spt4/5 and Elf1 on Pol II transcription of the B-form CTG•CAG tract duplex; second, we evaluated the effect of Spt4/5 and Elf1 on Pol II transcription of the non-B-form slip-out structures that can be generated in a transcription-coupled manner. This analysis revealed the opposite roles of Spt4/5 in modulating transcriptional arrest induced by the B-form CTG•CAG tract and the non-B form slip-out structures, respectively. Spt4/5, individually or cooperatively with Elf1, promotes Pol II bypass of the B-form CTG•CAG tract but inhibits Pol II bypass of the non-B-form slip-out structures. Our results support the structure of CTG•CAG repeat as a ‘function switch’. By changing from the B-form conformation (duplex structure) into a non-B conformation (slip-out), it transforms Spt4/5 from a transcription elongation facilitator to a transcription elongation repressor.

## MATERIALS AND METHODS

### Protein expression and purification

Expression and purification of yeast Elf1 were performed essentially as previously described (19). Recombinant Spt4/5 was expressed and purified as described (26). *Saccharomyces cerevisiae* 10-subunit Pol II was purified essentially as previously described (27). Briefly, Pol II (with a protein A tag in the Rpb3 subunit) was purified by an IgG affinity column (GE Healthcare), followed by Hi-Trap Heparin (GE Healthcare) and Mono Q anion exchange chromatography columns (GE Healthcare). Recombinant His_6_-tagged Rpb4/7 heterodimer was purified from *Escherichia coli* by Ni-affinity chromatography followed by gel filtration as previously described (28).

### Generation of non-B-form slip-out DNA templates

Single-stranded DNA oligonucleotides were purchased from Integrated DNA Technologies (IDT). The sequences of these oligonucleotides were designed such that the TS (template strand) and NTS (non-template strand) were completely complementary except for the presence or absence of a slip-out DNA insert. Annealing was performed in 10 mM Tris-HCl (pH 7.5) and 5 mM MgCl_2_ with an equal molecular ratio of each complementary single-stranded oligonucleotide for 10 min at 95°C, followed by slow cooling (15 h) to 23°C and subsequent native PAGE purification. Annealing of the oligonucleotide strands resulted in 9-nt sticky ends for ligation to the downstream of the elongation complex. The DNA/RNA sequences used in this study are shown in Supplementary Table S1.

### Generation of slip-out DNA containing elongation complex

Pol II elongation complex (EC10) was assembled essentially as previously described (29). First, radiolabeled 10-mer RNA was annealed to the TS DNA, followed by incubation with Pol II for 10 min at room temperature (23°C) and then 2 min at 37°C. To this, biotin-labeled NTS DNA was added and incubated for 5 min at 37°C, followed by 20 min at room temperature (23°C). The assembled elongation complex was incubated with streptavidin magnetic beads (NEB) for 30 min at room temperature (23°C) and subsequently washed with elongation buffer (EB) (20 mM Tris-HCl (pH 7.5), 5 mM MgCl_2_, 40 mM KCl, 5 mM DTT). The immobilized elongation complex was ligated to the downstream slip-out DNA and washed two times with EB buffer. Next, Rpb4/7 was added to a final concentration of 5 μM and incubated for 15 min at 23°C to generate 12-subunit elongation complex. Then the 12-subunit elongation complex was washed three times with EB buffer to remove excess Rpb4/7.

### *In vitro* transcription assay

*In vitro* transcription was initiated by adding 1 mM rNTPs (or specifically indicated in the figure legends). The concentrations for Spt4/5 and Elf1 were 200 and 500 nM, respectively. Reactions were performed at 23°C, allowed to continue for the desired time point, and then quenched by adding equal volume of formamide loading buffer (90% formamide, 50 mM EDTA, 0.05% xylene cyanol and 0.05% bromophenol blue). Samples were boiled for 15 min at 95°C in formamide loading buffer, and the extended RNA was separated from the unextended RNA by denaturing PAGE (6 M urea). The gels were visualized by phosphorimaging and quantified using Image Lab software (Bio-Rad).

### Structural modeling analysis

The Pol II elongation complex structure and Pol II–Spt4/5 complex structure used for analysis are previously published and the corresponding PDB IDs are listed in the figure legend. To show Pol II pausing at site 1 (-20, 20 nt upstream of CAG slip-out), the downstream duplex DNA was extended with ideal B-form DNA in COOT. All structure figures were prepared by PyMOL (www.pymol.org).

## RESULTS

### Establish a defined reconstituted system to study Pol II transcription through CTG•CAG repeat DNA *in vitro*

**Figure 1.**
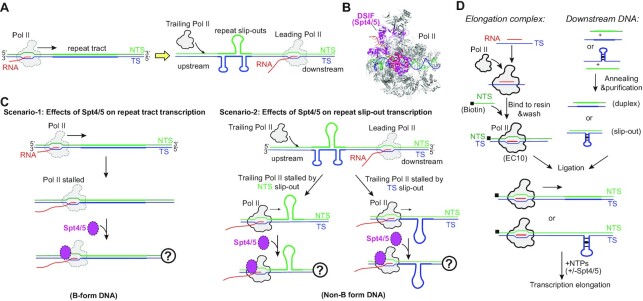
A reconstituted *in vitro* system to study Pol II transcription on CTG•CAG repeat DNA and roles of transcription factors. (**A**) The CTG•CAG trinucleotide repeat can form two different conformations: duplex tract form and the slip-out form that can be generated by separating the DNA duplex during transcription. (**B**) Structure of the Pol II elongation complex (Pol II EC) in complex with DSIF (human Spt4/5); PDB ID: 5OIK. (**C**) Two different scenarios to investigate the function of Spt4/5 on Pol II transcribing the CTG•CAG repeat. In scenario-1, it regulates Pol II bypass of the repeat tract duplex. In scenario-2, it regulates Pol II bypass of the slip-out DNA structures. (**D**) The primary experimental setup. Elongation complex and downstream DNA slip-out templates or repeat containing duplex were prepared separately and then ligated before adding NTPs to start the transcription. Transcription factors can be added to the system to evaluate their functions.

The CTG•CAG repeat has two possible conformations: one is the B-form duplex and the other is the non-B slip-out form. By separating the NTS and TS during transcription, it promotes the formation of slip-out structures in the CTG NTS and CAG TS. Once formed, NTS CTG slip-out and TS CAG slip-out structures impede transcription elongation of the trailing Pol II (Figure 1A). To evaluate the effect of Spt4/5 on Pol II transcription of CTG•CAG repeat (Figure 1B and C), we designed two different experimental settings to evaluate the effect of Spt4/5 on Pol II transcription of the B-form CTG•CAG tract duplex (scenario-1) or the non-B-form slip-out structures (scenario-2) (Figure 1C).

To achieve these goals, we reconstituted an *in vitro* transcription system using purified proteins from budding yeast and tested the effect of Spt4/5 on Pol II’s ability to bypass the CTG•CAG repeat tract or slip-out structures during transcription elongation. Briefly, we first ligated a pre-formed upstream Pol II elongation complex (EC) to a pre-formed downstream fragment containing either a B-form repeat tract duplex DNA or a non-B form site-specific slip-out structure. We then performed an *in vitro* transcription elongation assay using the newly generated scaffold in the presence or absence of Spt4/5 (Figure 1D). Compared with *in vivo* genetic study or *in vitro* study with nuclear extracts, this *in vitro* reconstituted system has several advantages: (i) The sequence and conformation of each strand of the DNA template is precisely defined. The conformation of DNA templates is defined and site-specific. In our reconstituted system, the pause inducing B-form CTG•CAG tract or non-B form slip-out DNA is located at a defined position of the DNA template and can be mapped at base resolution. (ii) The transcription machinery is biochemically purified with defined components. The purified Pol II EC is fully functional and faithfully recapitulates Pol II transcript elongation *in vivo*. (iii) The roles of Spt4/5 or other transcription factors can be directly dissected in this reconstituted system. Taken together, this defined reconstituted system allows us to directly dissect the roles of Spt4/5 in modulating CTG•CAG repeat induced Pol II transcription pausing/arrest and further explore the underlying mechanism.

### Spt4/5 promotes Pol II bypass of the B-form CTG•CAG repeat tract

**Figure 2.**
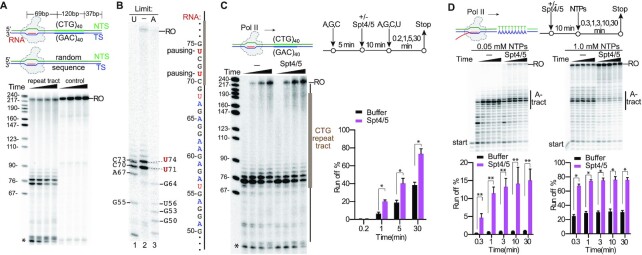
Spt4/5 is a positive elongation factor that promotes Pol II bypass of the CTG•CAG tract and poly-A tract duplex DNA. (**A**) The CTG•CAG tract induces transcription pausing. (**B**) Mapping the positions of Pol II paused on the CTG tract duplex DNA. RNA markers are generated by using limited UTP (2 μM, lane 1), or ATP (2 μM, lane 3). The transcript sequence is shown on right. (**C**) Spt4/5 promotes Pol II bypass of the CTG tract duplex DNA. (**D**) Spt4/5 promotes Pol II bypass of the poly-A tract duplex DNA. Markers shown on the left are a MspI digest of pBR322 plasmid. Asterisk indicates transcripts from reconstituted ECs that did not ligate to the downstream naked DNA.

We first tested the effect of B-form CTG•CAG repeat tract duplex on Pol II transcription elongation in our reconstituted system (Figure 2A). Consistent with previous results using nuclear extracts (23), we observed strong Pol II pausing induced by the B-form CTG•CAG repeat tract (Figure 2A). We mapped the pausing sites by comparing transcription products generated under saturating NTPs concentration (Figure 2B, lane 2) with the transcripts generated with limited UTP (Figure 2B, lane 1) or ATP (Figure 2B, lane 3), which would generate RNA transcript ladders of pausing sites before every U or A, respectively. This RNA ladder allows us to precisely map the positions of transcriptional pausing sites. We identified that pausing sites are located at the entrance of the B-form CTG repeat tract at positions U71 and U74 (Figure 2B), which is fully consistent with the previous study (23).

Previous structural studies revealed that elongation factor Spt4/5 interacts with Pol II stalk (Rpb4/7 subunits), clamp, RNA and upstream DNA to maintain the transcription bubble, position upstream DNA and retain the RNA transcript in the exit tunnel (Supplementary Figure S1; 19,30). To further dissect the functions of Spt4/5 in 12-subunit Pol II (WT) transcription of the B-form CTG repeat tract, we performed the transcription assay in the presence of Spt4/5. Strikingly, the addition of Spt4/5 greatly promotes 12-subunit Pol II bypass of the pausing sites and increases the run-off products (Figure 2C). We then compared the effects of Spt4/5 on 12-subunit Pol II versus 10-subunit Pol II (without the stalk) transcription through the B-form CTG•CAG repeat tract. Intriguingly, we found that Spt4/5 fails to promote 10-subunit Pol II bypass of the pausing at the B-form CTG•CAG repeat tract (Supplementary Figure S1), indicating the Pol II stalk is critical for the positive function of Spt4/5 on transcription elongation. These results provide strong evidence to support that Spt4/5, as a positive transcription elongation factor, directly facilitates the transcription of CTG•CAG repeat-containing gene (17). To further test whether the positive functions of Spt4/5 on transcription elongation on repetitive DNA sequences are a universal feature of Spt4/5, we also assessed its effect on Pol II transcription of a poly A-tract pausing sequence. Our results showed Spt4/5 significantly promotes Pol II bypass of the A-tract pausing and increases the run-off product in a similar manner (Figure 2D). Taken together, our results support the role of Spt4/5 as a universal positive elongation factor in rescuing transcriptional pausing from the repetitive duplex DNA sequences (B-form).

Elf1 works together with Spt4/5 to facilitate Pol II bypass of nucleosomal barriers, ensuring high transcription processivity on chromatin template (12). It is of great interest to test whether Elf1 and Spt4/5 can work together to facilitate Pol II bypass of B-form CTG•CAG repeat tract. Interestingly, we found that while Elf1 and Spt4/5 can work together to facilitate Pol II bypass of B-form CTG•CAG repeat tract, Elf1 alone fails to promote transcription bypass of CTG•CAG repeat tract (Supplementary Figure S2).

### Strand-specific transcriptional pausing by non-B form slip-out structures

**Figure 3.**
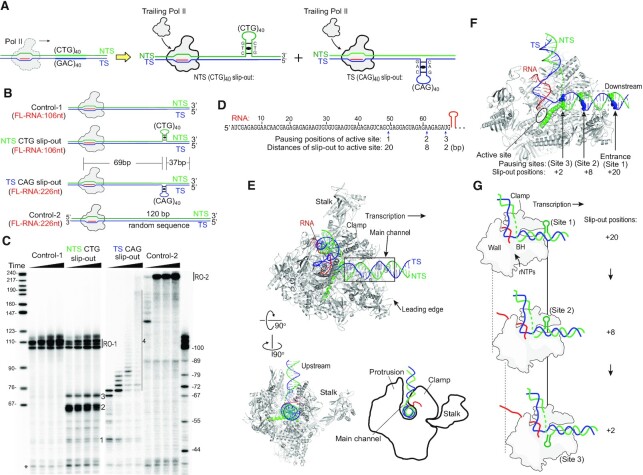
Analysis of the elongation complex paused on NTS CTG slip-out and TS CAG slip-out templates. (**A**) Scheme of transcription of the CTG•CAG tract induces the NTS CTG slip-out and TS CAG slip-out formation. (**B**) Diagram of the position of the slip-out structures in the DNA substrates. The size of the expected full-length RNA is indicated. (**C**) Mapping of RNA Pol II transcription blockage by the slip-out structures. Three distinct pausing sites before the hairpin runs into the active site (expected RNA size is 69 nt) were labeled. Markers on the left are a MspI digest of pBR322 plasmid. Markers on the right are different lengths of the transcript RNA, which are generated by Pol II transcription using the same DNA template but with limited UTP concentration to induce Pol II pausing. Asterisk indicates transcripts from reconstituted ECs that did not ligate to the downstream naked DNA. (**D**) The sequence of the transcript RNA before the slip-out structure region is shown on the top. The 3′ of the truncated RNA transcript and the distance of the slip-out structure to the active site when Pol II pauses are shown on the bottom. (**E**) The main channel of Pol II EC holds the downstream DNA. The PDB ID for Pol II EC elongation complex structure is 5FLM. The position of the downstream dsDNA outside of Pol II binding region was extended by coot with ideal B form duplex. (**F**) Positions of the slip-out structure when Pol II stalls. During transcription elongation, the main channel entrance edge detects the hairpin structure first and results in weak pausing (site 1), and then Pol II translocates forward and pauses at site 2, where the Pol II active site is at 8 nt upstream of the hairpin. Pol II further pauses when the hairpin runs close to the active site (site 3, where the Pol II active site is at 2–3 nt upstream of the hairpin). The main channel is shown in the dashed rectangle. (**G**) Diagram of the positions of slip-out when Pol II is stalled by these structures. The positions of NTS slip-out induced Pol II pausing are shown here.

The CTG•CAG tract DNA has been shown to form CTG and CAG slip-out structures (21,22), and these DNA slip-out structures induce Pol II pausing in nuclear extract (8). To further understand the underlying mechanism of Pol II pausing/arrest induced by these non-B form slip-out structures, we set out to characterize Pol II transcription on the NTS CTG slip-out and TS CAG slip-out (Figure 3A). In addition, we generated two controls for comparison (Figure 3B): control-1 has a short template strand (identical to that of the NTS CTG slip-out scaffold) and a fully complementary NTS to form B-form duplex; control-2 has the same length of long template strand (as that of the TS CAG slip-out scaffold), containing a 120 bp random sequence B-form duplex DNA instead of the CAG slip-out. Thus, the run-off transcript length from control-1 is expected to be the same as the run-off transcript from the NTS CTG slip-out, whereas the run-off transcript length from control-2 is expected to be the same as the run-off transcript from the TS CAG slip-out (Figure 3B and C).

Our results showed the NTS CTG slip-out generates one minor and two major pausing products, labeled as 1, 2 and 3, respectively, in addition to the run-off products (Figure 3C). To map the pausing positions, we generated an RNA marker and identified the length of these three pausing products induced by NTS slip-out, which are ∼49, 61 and 67 nt, respectively (Figure 3C and D). Interestingly, the TS CAG slip-out has different effects on Pol II transcription. First, the TS CAG slip-out has a weaker pausing effect at pausing site 2, but induces Pol II pausing at multiple positions beyond 69 nt (labeled as area 4 in Figure 3C). Second, transcription of the TS slip-outs template produces much less overall run-off transcription product. We observed almost no run-off transcription product from a TS slip-out template, but only a modest reduction of run-off transcription product from an NTS slip-out template. This result indicates the TS slip-out has a stronger overall inhibitory effect than the NTS slip-out, which is consistent with the previous results (8).

To gain a better mechanistic understanding of how Pol II detects and interacts with the slip-out or hairpin structures, we mapped the pausing sites within the Pol II elongation complex structure (31; Figure 3D–F). As shown in Figure 3B and D, the distance between the RNA 5′ end and the 5′-position of CTG or CAG slip-out is 69 nt. Interestingly, the RNA transcripts at identified proximal pausing sites [positions 1 (-20, 20 nt upstream of CAG slip-out), 2 (-8, 8 nt upstream of CAG slip-out) and 3 (-2, 2 nt upstream of CAG slip-out)] were 2–20 nt shorter than 69 nt, respectively. These proximal pausing sites suggest Pol II can sense the slip-out structures and pause before these non-B form DNA structures reach the Pol II active site. Our structural analysis revealed that Pol II pauses at site 1 when the polymerase first encounters the slip-out structure, likely due to the initial interaction or steric clash of the leading edge of Pol II EC and the slip-out structure (Figure 3E and F). As Pol II translocates further downstream, the bulky slip-out structures enter the main channel of Pol II and may interact or clash with the polymerase (sites 2 and 3 in Figure 3E–G). We predict that there will also be conformational changes of the slip-out structure, and perhaps the Pol II clamp as well, to further avoid steric clash and to get better accommodated within the main channel. To proceed beyond 69 nt, the bulky slip-out structure needs to be deformed to allow Pol II forward translocation. Future studies would be needed to unravel the structural details of the interactions between slip-out structures and Pol II residues within the main channel.

### Spt4/5 and Elf1 prevent, rather than stimulate, transcriptional bypass of non-B form slip-out structures *in vitro*

**Figure 4.**
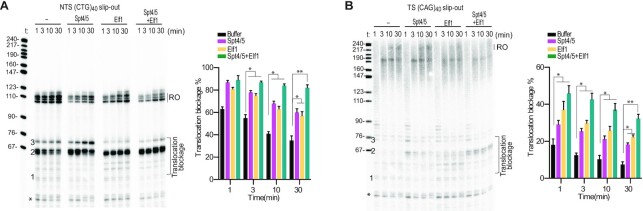
Spt4/5 and Elf1 inhibit transcriptional bypass of the slip-out structures. (**A**) Spt4/5 and Elf1 inhibit transcriptional bypass of NTS CTG slip-out individually and synergistically. (**B**) Spt4/5 and Elf1 inhibit transcriptional bypass of TS CAG individually and synergistically. Data are means ± SEM, *n* = 3 (*n* = 3, two-tailed Student’s *t*-test, *P < 0.05, **P < 0.01). Asterisk indicates transcripts from reconstituted ECs that did not ligate to the downstream naked DNA.

Previous study revealed that Spt4/5 and Elf1 can facilitate Pol II bypass of nucleosomal barriers (12). This observation motivates us to test whether Spt4/5 and Elf1 can also facilitate Pol II bypass of non-B slip-out structures. Surprisingly, we found Spt4/5 and Elf1 inhibit, rather than stimulate, Pol II transcriptional bypass of NTS slip-out structures. As shown in Figure 4A, Spt4/5 and Elf1 prevent Pol II transcriptional bypass of a NTS CTG slip-out containing scaffold. Specifically, the addition of Spt4/5 enhances Pol II pausing/arrest at site 2 and site 3 (Figure 4A). Similarly, the addition of Elf1 greatly enhances Pol II pausing/arrest at site 2 (Figure 4A). Importantly, when both Spt4/5 and Elf1 are present, transcription pausing/arrest at site 2 dramatically increases, and the run-off product is greatly reduced, indicating a synergistic effect between Spt4/5 and Elf1 (Figure 4A and Supplementary Figure S3).

Next, we evaluated the effect of Spt4/5 and Elf1 on Pol II transcription of TS CAG slip-out (Figure 4B). We observed that Spt4/5 and Elf1 both individually and cooperatively slow down Pol II bypass of TS CAG slip-out. Of note, consistent with previous study using nuclear extracts, the TS CAG slip-out shows a much weaker inhibitory effect at the proximal pausing sites (sites 1, 2 and 3) than the NTS CTG slip-out (8). Together, our results reveal that elongation factors Spt4/5 and Elf1 individually and cooperatively prevent transcriptional bypass of both NTS CTG slip-out and TS CAG slip-out.

### Spt4/5 and Elf1 prevent transcriptional bypass of non-B form perfect hairpin

**Figure 5.**
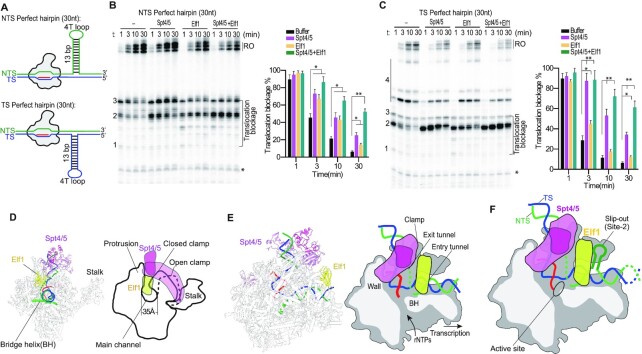
Spt4/5 and Elf1 inhibit transcriptional bypass of a perfect hairpin. (**A**) Scheme of the perfect hairpin templates. (**B**) Spt4/5 and Elf1 inhibit transcriptional bypass of NTS perfect hairpin individually and synergistically. (**C**) Spt4/5 and Elf1 inhibit transcriptional bypass of TS perfect hairpin individually and synergistically. (**D**) Spt4/5 and Elf1 stabilize Pol II in a closed conformation. The width of the main channel is about 35 Å. (**E**) Structure and cartoon of the Spt4/5-Elf1-Pol II complex; PDB ID: 5XON. For simplicity, the downstream nucleosome was omitted. (**F**) Analysis of the slip-out induced pausing in the structure of Spt4/5-Elf1-Pol II complex. NTS induced Pol II pausing at position 2 is shown as an example to explain how Spt4/5-Elf1 prevents Pol II bypass of the slip-out structures. Data are means ± SEM (*n* = 3, two-tailed Student’s *t*-test, *P < 0.05, **P < 0.01).

To address whether Spt4/5 and Elf1 also have inhibitory effect on Pol II transcription of other non-B form slip-out structures, we tested the effect of Spt4/5 and Elf1 on Pol II transcription of perfect hairpin structures on NTS or TS (Figure 5A). The designed perfect hairpin contains a 13 base pair stem and a 4 nt T loop, which has the same total length of (CAG)_10_ slip-out (Supplementary Table S1 and Figure S4). Similar to their inhibitory effect on NTS CTG slip-out structures (shown in Figure 4), Spt4/5 and Elf1 both individually and cooperatively inhibit Pol II bypass of the NTS perfect hairpin structure (Figure 5B) as well as the TS perfect hairpin (Figure 5C). Our data further validated Spt4/5 and Elf1 inhibit transcriptional bypass of the (CAG)_10_ slip-out structures in a similar manner (Supplementary Table S1 and Figure S4). Taken together, we showed that Spt4/5 and Elf1 can greatly inhibit Pol II bypass of non-B form CAG/CTG repeat slip-out and/or non-B form perfect hairpin DNA structures.

Why do Spt4/5 and Elf1 have an inhibitory, instead of stimulatory, role in regulating the transcriptional bypass of the non-B slip-out structures and perfect hairpin? Previous structural studies found Spt4/5 and Elf1 stabilize Pol II in a closed conformation by encircling both the upstream and downstream DNA (19,30) (Figure 5D). We then analyzed the pausing sites in the context of Pol II elongation complex bound with Spt4/5 and Elf1 (Figure 5E,F). In the Spt4/5-Elf1-Pol II structures, Spt4/5 forms multiple interactions with the Pol II clamp, protrusion, stalk, wall and dock domains. The Spt5-NGN domain binds between the clamp and the protrusion, occupying the space between the clamp (Rpb1 coiled-coil) and core (Rpb2 protrusion and lobe) modules (Figure 5E). Elf1 is located between the Rpb2 lobe domain and the Rpb1 clamp-head domain of Pol II, bridging the Pol II central cleft. Thus, Spt5-NGN-KOW1, Spt4, Rpb2 protrusion and wall establish a ‘DNA exit tunnel (upstream DNA)’, whereas Elf1, Rpb2 lobe and Rpb1 clamp head compose a ‘DNA entry tunnel (downstream DNA)’ (Figure 5E) (19).

The size of a slip-out or a perfect hairpin non-B form structure can be larger than the width of the main channel of Pol II (Figure 5D, about 35 Å). For example, the dimension of a perfect hairpin (11 bp and a 5 nt loop) is about 20 Å × 20 Å × 40 Å (32). As a result, the perfect hairpin structure will clash with the DNA ‘entry tunnel’ and ‘exit tunnel’ when it run into the main channel. During the translocation of Spt4/5-Elf1-Pol II complex on the slip-out DNA containing templates, the slip-out DNA will encounter Elf1 first and cause a strong steric clash. Because Elf1 is localized close to the pausing site 2, it increases transcriptional stalling at site 2 (Figure 5F). Similarly, because Spt4/5 bridges Pol II main channel and stabilizes Pol II in a closed conformation, the slip-out structures will collide with Spt4/5 during transcription (Figure 5F). Importantly, since Spt4/5 and Elf1 cooperatively bind to Pol II (19), both together will stabilize the interaction between Spt4/5-Elf1 and Pol II and thus enhance pausing at site 2. Consistent with these observations, we found the inhibitory effect of Spt4/5 and Elf1 is dependent on their binding to Pol II EC, as the mutants that weaken the interactions with Pol II EC abolish the inhibitory effects of Spt4/5 and Elf1 (Supplementary Figure S5).

## DISCUSSION

### Transcriptional arrest induced by CTG•CAG tract duplex and slip-out structures

Much of the current understanding of CTG repeat-induced Pol II pausing is derived from studies using nuclear extracts or cell-based assays (8,23,33). These assays are valuable but cannot be used to elucidate the complete details of the biochemical mechanisms due to the undefined mix of proteins, nucleic acids and biochemicals in the assay system. The effects of CTG•CAG repeat tract on the activity of purified transcription machinery were obtained from the single subunit viral T7 RNA polymerase (34,35), which is structurally unrelated to Pol II. The underlying mechanism of CTG or CAG repeat slip-out induced Pol II transcriptional pausing and the functions of transcription factors on the regulation of Pol II transcriptional pausing remains elusive. Therefore, reconstituting a defined system for eukaryotic Pol II transcription and analyzing the roles of individual transcription factors in Pol II pausing/processing of the CTG•CAG repeat region is in an urgent need. Here we reconstituted Pol II transcription of the CTG•CAG tracts and CTG, CAG slip-outs entirely from purified proteins and DNA/RNA scaffolds. This system faithfully recapitulates Pol II transcription on CAG containing DNA templates that allows us to dissect the molecular mechanisms that cause Pol II pausing/arrest and investigate the functions of transcription factors in rescuing transcriptional pausing/arrest of the repeat tract or repeat slip-out structures.

We studied the effect of the CTG•CAG repeat tract and slip-out DNA structures on Pol II elongation. In line with previous study using nuclear extract as a source of transcription machinery (23), we showed Pol II is arrested upon encountering a CTG•CAG tract duplex. Importantly, we uncovered Spt4/5 significantly promotes Pol II bypass of the CTG•CAG tract and increases the run-off product. As a further validation, we showed that Spt4/5 effectively promotes Pol II bypass of poly-A tract pausing sequence. Together, our biochemical results show Spt4/5 function as a positive elongation factor that facilitates Pol II transcription of B-form CTG•CAG tract and poly-A tract duplex DNA.

We further studied the effect of slip-out DNA structures, which can be generated by the transcription of CTG•CAG tract DNA, on Pol II transcription elongation. Intriguingly, we found the pausing/stall patterns induced by NTS CTG slip-out and TS CAG slip-out on transcription are quite different. The NTS CTG slip-out induces strong Pol II pausing at three different sites before the slip-out structure runs into the Pol II active site. The TS CAG slip-out has a weaker pausing effect at these sites. However, it induces strong transcription pausing after Pol II runs into the split-out structures (area 4 in Figure 3C).

Our structural analysis further revealed that slip-out structures induce polymerase pausing at three sites by interacting with the Pol II main channel. The first slip-out structure induced weak transient pausing is due to the interaction with the Pol II leading edge of the main channel, whereas the latter two arrests are attributed to a direct steric clash with the main channel of Pol II, indicating the main channel is responsible for sensing bulky non-B DNA structures. Thus, in addition to its ability in recognizing DNA lesions (36), epigenetic modifications (37) and DNA minor groove binding molecules (38), Pol II can recognize bulky non-B DNA structures.

### Dual Roles of Spt4/5 and Elf1 in transcriptional processing of CTG repeats

**Figure 6.**
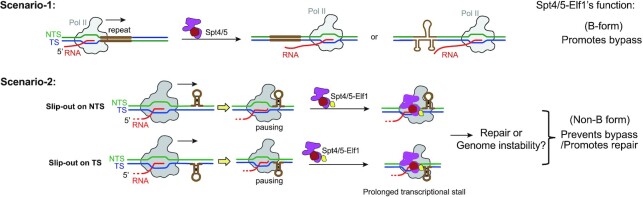
Model of the functions of Spt4/5 and Elf1 in transcription of CTG•CAG repeat. In scenario-1, as a transcription elongation facilitator, Spt4/5 or the Spt4/5–Elf1 complex promotes Pol II bypass of the CTG•CAG repeat tract duplex. In scenario-2, Spt4/5 and Elf1 inhibit Pol II bypass of the NTS and TS slip-out. Spt4/5 and Elf1 facilitate the detection of non-B DNA structure and may promote non-B DNA repair.

Spt4/5 have been shown to facilitate the protein expression of CTG•CAG repeat-containing gene in cell lines (16,17) as well as in mice (39). Deletion of Spt4/Supt4 reduces mHTT protein in neuronal cells and decreases its aggregation and toxicity (17,39). Importantly, a direct connection between Spt4/5 regulated transcription elongation and protein expression was lacking. Here, we showed that Spt4/5 plays a positive role in promoting Pol II bypass of the B-form CTG•CAG tract (Figure 6), which is fully consistent with their *in vivo* functions as elongation factors that increase Pol II processivity and facilitate the expression of CTG repeat-containing gene (14). This positive elongation factor function of Spt4/5 might also be applied to other repeated DNA transcription, such as GGGGCC hexanucleotide repeat in C9orf72 (40). Interestingly, it is worth noting that although genetic study indicates Elf1 is an elongation factor, our data (Supplementary Figure S2) and previous biochemical study found Elf1 alone is not able to help Pol II bypass pausing sites *in vitro* (19). One possible explanation for the difference between the *in vivo* and the *in vitro* observation is the *in vivo* genome is much longer than the DNA used *in vitro* study. In line with this, the transcription of longer genes is more sensitive to Elf1 knockout than that of the shorter genes (41).

However, in contrast to their positive effect on CTG•CAG tract transcription, we uncovered that Spt4/5 greatly inhibits Pol II transcription elongation on the slip-out DNA template, which can be generated by transcription of the CTG•CAG tract (Figure 6). This is in sharp contrast to the canonical roles of Spt4/5 as a positive transcription elongation factor that increases the processivity of Pol II and promotes transcription of nucleosome or naked DNA (10). The inhibitory effect of Spt4/5 on Pol II transcription of slip-out DNA structures leads to persistent transcriptional arrest, which in turn may result in transcription–transcription and transcription–replication conflicts or act as a signal to trigger transcription-coupled nucleotide excision repair (TC-NER) (36). The subsequent TC-NER of CAG slip-out structures has been proposed to contribute to CTG or CAG repeat instability (29,42).

Elf1 is a transcription elongation factor (18) that binds to the main channel of Pol II (19) and also plays a critical role in facilitating TC-NER (43). Our biochemical study and structural analysis show it prevents Pol II from bypassing the slip-out structures by circulating the Pol II main channel and forming a very narrow entry tunnel for the downstream DNA duplex. This result indicates that Elf1 has a previously overlooked function. Elf1 alone, or in joint action with Spt4/5, is able to detect the secondary structures (non-B DNA structure) in the downstream DNA template during transcription. This may also facilitate Pol II to recognize DNA lesions that may disrupt the canonical B-form duplex structure and consequently regulate TC-NER. The function of Elf1 identified here is an important direction for future studies. The role of Elf1/ELOF1 in CTG or CAG repeat instability *in vivo* awaits to be examined.

Taken together, this study greatly expands our understanding of the functional roles of Spt4/5 and Elf1 as well as their roles in modulating transcription pausing/bypass of trinucleotide repeats. In contrast to canonical views of Spt4/5 and Elf1 as positive transcription elongation factors, we reveal that dual functions of Spt4/5 and Elf1 depend on the context and structures of the DNA template. On one hand, we showed that Spt4/5 and Elf1 play a canonical, positive role in stimulating transcription elongation on B-form triplet repeat DNA duplex. This is consistent with the reported role of Spt4/5 in the expression of facilitated triplet repeat coded toxic proteins, such as HTT protein (17,39). On the other hand, the formation of non-B structures of CAG repeat converts Spt4/5 and Elf1 from a positive transcription elongation factor to a negative transcription factor and leads to prolonged transcriptional arrest. Therefore, we uncovered important functional interplays between template DNA structure and the function of transcription factors. Our studies suggest that Spt4/5 and Elf1 have an additional role in helping Pol II to discriminate downstream DNA structures (B-form versus non-B-form).

The prolonged transcriptional arrest induced by non-B-form slip-out/hairpin may have implications with transcription-coupled CTG/CAG repeat fragility, instability and repair (20). Stalled transcription complexes can serve as a signal to directly trigger downstream DNA repair pathways, such as TC-NER (9,20). Alternatively, transcription arrest induces replication–transcription collision and causes a DNA nick or a double-strand break (DSB), or R-loop formation (20). These events can trigger multiple DNA repair pathways (20), including DSB repair pathways (such as nonhomologous end joining (NHEJ) and microhomology-mediated end joining (MMEJ)), mismatch repair (MMR), base excision repair (BER) and nucleotide excision repair (NER). By regulating transcription bypass/arrest, Spt4/5 and Elf1 may also have potential roles in modulating trinucleotide repeat DNA stability and downstream repair pathways. Moreover, given the modular structure of Spt5 (10), Spt5 may serve as a bridge between the elongating RNA polymerase and other cellular factors during this process. In addition, Elf1 is recently identified as a novel core transcription-coupled repair factor for canonical TC-NER (41,43). It would be interesting to investigate the roles of Spt4/5, Elf1 or other elongation factors in modulating transcription-coupled trinucleotide repeat fragility, instability and the choice of repair pathways in the future.

## Supplementary Material

gkab240_Supplemental_File
