# Supplementary material for: Opposite roles of transcription elongation factors Spt4/5 and Elf1 in RNA polymerase II transcription through B-form versus non-B DNA structures
Source: Nucleic Acids Res. 2021 Apr 20;49(9):4944–53. doi: 10.1093/nar/gkab240 (PMC8136819; doi:10.1093/nar/gkab240)
Supplement: gkab240_Supplemental_File [file gkab240_supplemental_file.pdf]

Opposite roles of transcription elongation factors Spt4/5 and Elf1 on RNA polymerase II transcription through B-form versus non-B DNA structures

Jun Xu[1], Jenny Chong[1], Dong Wang[1,2,3,*]

[1]Division of Pharmaceutical Sciences, Skaggs School of Pharmacy & Pharmaceutical Sciences; University of California, San Diego, La Jolla, California 92093, United States

[2]Department of Cellular and Molecular Medicine, University of California, San Diego, La Jolla, California 92093, United States

[3]Department of Chemistry and Biochemistry, University of California, San Diego, La Jolla, California 92093, United States

SUPPORTING INFORMATION

Table of contents

Table S1. Sequences of the oligonucleotides.

1. **Figure S1.** Specific interactions between Spt4/5 and Pol II is critical for Spt4/5 facilitated elongation.
2. **Figure S2**. Spt4/5-Elf1, but not Elf1 alone, can promote Pol II bypass of the B-form CTG•CAG tract.
3. **Figure S3.** Synergistic effect of Spt4/5 and Elf1 on preventing Pol II bypass of the slip-out structure.
4. **Figure S4.** Spt4/5 and Elf1 inhibit transcriptional bypass of the $(CAG)_{10}$ slip-out structures.
5. **Figure S5.** Inhibition of Pol II bypass of $(CAG)_{10}$ slip-out by Spt4/5 and Elf1 is dependent on the Spt4/5-Pol II interaction and Elf1-Pol II interaction.

Table S1. Sequences of the oligonucleotides.

Figure	Pa<br>nel	Oligo sequence: (All oligos are in 5’ to 3’ direction. Repeat regions are highlighted in orange, and perfect hairpins sequences are underlined and bolded. RNA binding site on TS is shown in bolded red).
Figure 2	A,<br>B,<br>C	**NTS:** Biotin-TTTCTATATTGACTGAGTTCAGCTCATATTTATCGAGAGGAACAACGAGAGAGAGAAGTGTGTGAAGTGAGAGAG<br>TCAGCGAGGAGTAGAGAAGGAGATGCTGCTGCTGCTGCTGCTGCTGCTGCTGCTGCTGCTGCTGCTGCTGCTGCTGCTGCTGCTGCTGC<br>TGCTGCTGCTGCTGCTGCTGCTGCTGCTGCTGCTGCTGCTGCTGCTGCTGCTGCTGGAGAGGAGAGAGAGAAGAGAAGAGAGAGGCATG<br>TCGG<br>**TS:** CCGACATGCCTCTCTCTTCTCTTCTCTCTCTCCTCTCCAGCAGCAGCAGCAGCAGCAGCAGCAGCAGCAGCAGCAGCAGCA<br>GCAGCAGCAGCAGCAGCAGCAGCAGCAGCAGCAGCAGCAGCAGCAGCAGCAGCAGCAGCAGCAGCAGCAGCAGCAGCATCTCCTTCTCT<br>ACTCCTCGCTGACTCTCTCACTTCACACACTTCTCTCTCTCGTTGT**TCCTCTCGAT**AAATATGAGCTGAACTCAGTCAATATAG<br>RNA: AUCGAGAGGA
	D	**NTS:** Biotin-TGAAGAATCATGCTTTCCTTGGTCAGATATGATATCCTTATATTCATCGAGAGGAATAGTAGAGTTTTTTTTTC<br>CAACAGATGTGCAGAC<br>**TS:** GTCTGCACATCTGTTGGAAAAAAAAACTCTACTATTCCTCTCGATGAATATAAGGATATCATATCTGACCAAGGAAAGCA<br>TGATTCT<br>**RNA:** UUUUUUUUUUUUUAUCGAGAGG
Figure 3	A,<br>B,<br>C	**Control-1**<br>**NTS:**Biotin-TTTCTATATTGACTGAGTTCAGCTCATATTTATCGAGAGGAACAACGAGAGAGAGAAGTGTGTGAAGTGAGAGAGT<br>CAGCGAGGAGTAGAGAAGGAGATGGAGAGGAGAGAGAGAAGAGAAGAGAGAGGCATGTCGG<br>**TS:** CCGACATGCCTCTCTCTTCTCTTCTCTCTCTCCTCTCCATCTCCTTCTCTACTCCTCGCTGACTCTCTCACT<br>TCACACACTTCTCTCTCTCGTTGT**TCCTCTCGAT**AAATATGAGCTGAACTCAGTCAATATAG<br>**RNA:** AUCGAGAGGA<br><br>**NTS CTG slip-out**<br>**NTS:**Biotin-TTTCTATATTGACTGAGTTCAGCTCATATTTATCGAGAGGAACAACGAGAGAGAGAAGTGTGTGAAGTGAGAGAGTC<br>AGCGAGGAGTAGAGAAGGAGATGCTGCTGCTGCTGCTGCTGCTGCTGCTGCTGCTGCTGCTGCTGCTGCTGCTGCTGCTGCTGCTGCTG<br>CTGCTGCTGCTGCTGCTGCTGCTGCTGCTGCTGCTGCTGCTGCTGCTGCTGCTGCTGGAGAGGAGAGAGAGAAGAGAAGAGAGAGGCATGTC<br>GG<br>**TS:** CCGACATGCCTCTCTCTTCTCTTCTCTCTCTCCTCTCCATCTCCTTCTCTACTCCTCGCTGACTCTCTCACTTCACACACTT<br>CTCTCTCTCGTTGT**TCCTCTCGAT**AAATATGAGCTGAACTCAGTCAATATAG<br>**RNA:** AUCGAGAGGA<br><br>**TS CAG slip-out**<br>**NTS:**Biotin-TTTCTATATTGACTGAGTTCAGCTCATATTTATCGAGAGGAACAACGAGAGAGAGAAGTGTGTGAAGTGAGAGAGTC<br>AGCGAGGAGTAGAGAAGGAGATGGAGAGGAGAGAGAGAAGAGAAGAGAGAGGCATGTCGG<br>**TS:** CCGACATGCCTCTCTCTTCTCTTCTCTCTCTCCTCTCCAGCAGCAGCAGCAGCAGCAGCAGCAGCAGCAGCAGCAGCAGCAG<br>CAGCAGCAGCAGCAGCAGCAGCAGCAGCAGCAGCAGCAGCAGCAGCAGCAGCAGCAGCAGCAGCAGCAGCAGCAGCATCTCCTTCTCTA<br>CTCCTCGCTGACTCTCTCACTTCACACACTTCTCTCTCTCGTTGT**TCCTCTCGAT**AAATATGAGCTGAACTCAGTCAATATAG<br>**RNA:** AUCGAGAGGA<br><br>**Control-2**<br>**NTS:** Biotin-TTTCTATATTGACTGAGTTCAGCTCATATTTATCGAGAGGAACAACGAGAGAGAGAAGTGTGTGAAGTGAGAGAG<br>TCAGCGAGGAGTAGAGAAGGAGATGGAGCTCGGTACCCGGGGATCCTCTAGAGTGGGAGCTCGGAACACTATCCGACTGGCACCGGCAA<br>GGTCGCTGTTCAATACATGCACAGGATGTATATATCTGACACGTGCCTGGAGACTAGAGAGGAGAGAGAGAAGAGAAGAGAGAGGCATG<br>TCGG<br>**TS:** CCGACATGCCTCTCTCTTCTCTTCTCTCTCTCCTCTCTAGTCTCCAGGCACGTGTCAGATATATACATCCTGTGCATGTATT<br>GAACAGCGACCTTGCCGGTGCCAGTCGGATAGTGTTCCGAGCTCCCACTCTAGAGGATCCCCGGGTACCGAGCTCCATCTCCTTCTCTA<br>CTCCTCGCTGACTCTCTCACTTCACACACTTCTCTCTCTCGTTGT**TCCTCTCGAT**AAATATGAGCTGAACTCAGTCAATATAG<br>**RNA:** AUCGAGAGGA
Figure 4	A	**NTS CTG slip-out**<br>**NTS:** Biotin-TTTCTATATTGACTGAGTTCAGCTCATATTTATCGAGAGGAACAACGAGAGAGAGAAGTGTGTGAAGTGAGAGAGTC<br>AGCGAGGAGTAGAGAAGGAGATGCTGCTGCTGCTGCTGCTGCTGCTGCTGCTGCTGCTGCTGCTGCTGCTGCTGCTGCTGCTGCTGCTG<br>CTGCTGCTGCTGCTGCTGCTGCTGCTGCTGCTGCTGCTGCTGCTGCTGCTGCTGCTGGAGAGGAGAGAGAGAAGAGAAGAGAGAGGCATGTC<br>GG<br>**TS:** CCGACATGCCTCTCTCTTCTCTTCTCTCTCTCCTCTCCATCTCCTTCTCTACTCCTCGCTGACTCTCTCACTTCACACACTT<br>CTCTCTCTCGTTGT**TCCTCTCGAT**AAATATGAGCTGAACTCAGTCAATATAG<br>**RNA:** AUCGAGAGGA

	B	**TS CAG slip-out**<br>**NTS:** Biotin-TTTCTATATTGACTGAGTTCAGCTCATATTTATCGAGAGGAACAACGAGAGAGAGAAGTGTGTGAAGTGAGAGAGTC<br>AGCGAGGAGTAGAGAAGGAGATGGAGAGGAGAGAGAGAAGAGAAGAGAGAGGCATGTCGG<br>**TS:** CCGACATGCCTCTCTCTTCTCTTCTCTCTCTCCTCTCCAGCAGCAGCAGCAGCAGCAGCAGCAGCAGCAGCAGCAGCAGCAG<br>CAGCAGCAGCAGCAGCAGCAGCAGCAGCAGCAGCAGCAGCAGCAGCAGCAGCAGCAGCAGCAGCAGCAGCAGCAGCATCTCCTTCTCTA<br>CTCCTCGCTGACTCTCTCACTTCACACACTTCTCTCTCTCGTTGT**TCCTCTCGAT**AAATATGAGCTGAACTCAGTCAATATAG<br>**RNA:** AUCGAGAGGA
Figure 5	B, C	**NTS perfect hairpin:**<br>**NTS:** Biotin-TTTCTATATTGACTGAGTTCAGCTCATATTTATCGAGAGGAACAACGAGAGAGAGAAGTGTGTG<br>AAGTGAGAGAGTCAGCGAGGAGTAGAGAAGGAGATG**GCCACGGACGGCTTTTGCCCGTCCGTGGC**GAGAGGAGAGAGAGAAGAGAAGA<br>GAGAGGCATGTCGG<br>**TS:** CCGACATGCCTCTCTCTTCTCTTCTCTCTCTCCTCTCCATCTCCTTCTCTACTCCTCGCTGACTCTCTC<br>ACTTCACACACTTCTCTCTCTCGTTGT**TCCTCTCGAT**AAATATGAGCTGAACTCAGTCAATATAG<br>**RNA:** AUCGAGAGGA<br><br>**TS perfect hairpin:**<br>**NTS:** Biotin-TTTCTATATTGACTGAGTTCAGCTCATATTTATCGAGAGGAACAACGAGAGAGAGAAGTGTGTG<br>AAGTGAGAGAGTCAGCGAGGAGTAGAGAAGGAGATGGAGAGGAGAGAGAGAAGAGAAGAGAGAGGCATGTCGG<br>**TS:** CCGACATGCCTCTCTCTTCTCTTCTCTCTCTCCTCTC**CGGTGCCTGCCCGTTTTCGGGCAGGCACCG**CA<br>TCTCCTTCTCTACTCCTCGCTGACTCTCTCACTTCACACACTTCTCTCTCTCGTTGT**TCCTCTCGAT**AAATATGAGCTGAACTCAGTCA<br>ATATAG<br>**RNA:** AUCGAGAGGA

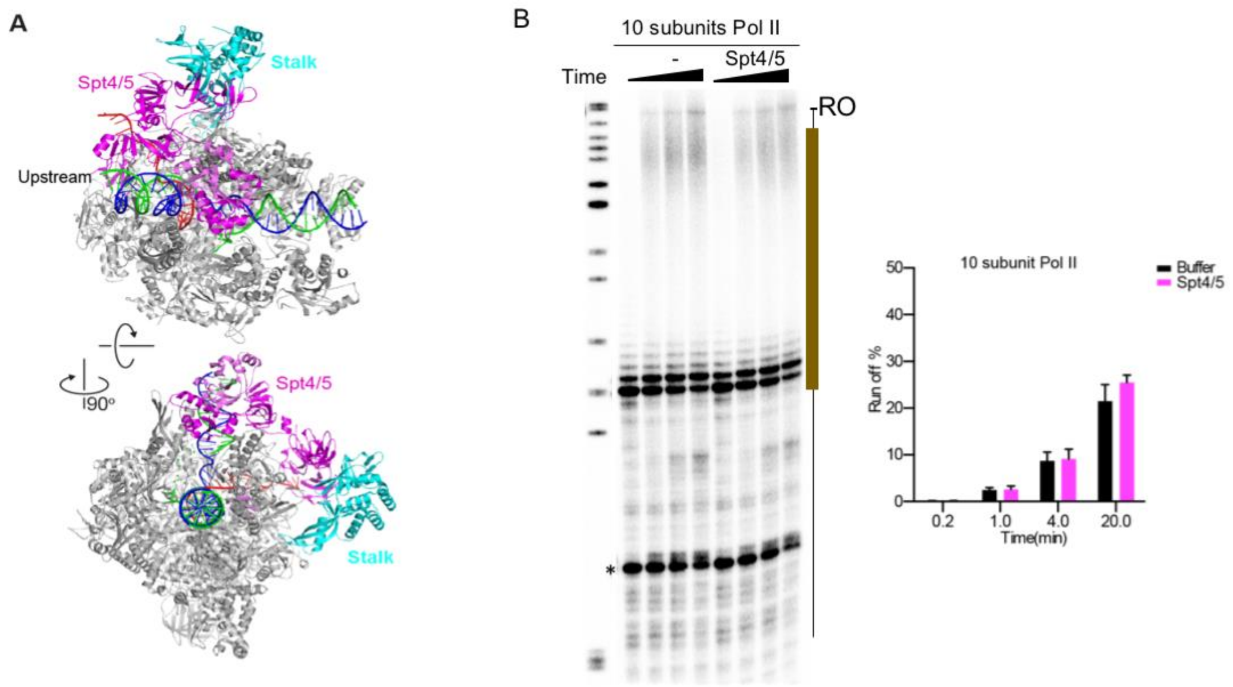

Figure S1.
Specific interaction between Spt4/5 and Pol II is critical for Spt4/5 facilitated elongation. (A) The stalk of Pol II is essential for Spt4/5 recruitment. PDB ID: 5OIK. (B) The stalk is critical for Spt4/5 facilitated Pol II elongation. The same DNA template in Figure 2 is used here.

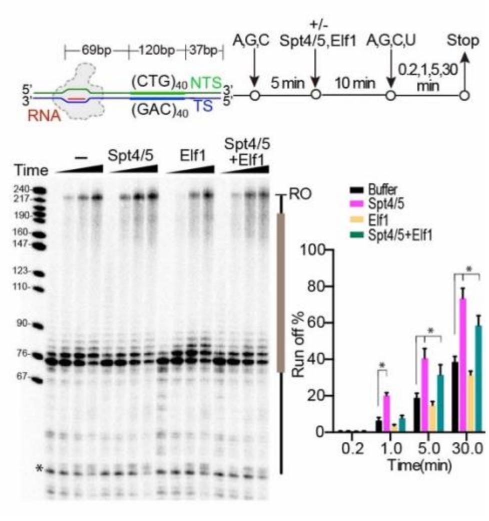

Figure S2. Spt4/5-Elf1, but not Elf1 alone, can promote Pol II bypass of the CTG•CAG repeat tract. Part of the figure is shown in Figure 2C.

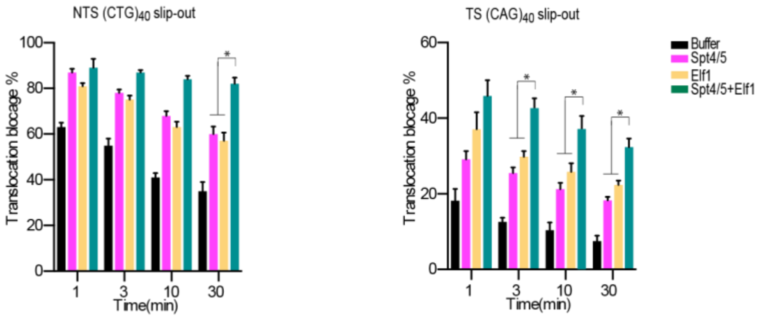

Figure S3. Synergistic effect of Spt4/5 and Elf1 on preventing Pol II bypass of the slip-out structure. The inhibitory effect of Spt4/5-Elf1 is significantly stronger than that of individual Spt4/5 or Elf1 at the last time point (30min) in both NTS and TS slip-outs. Data obtained from three independent experiments are shown as means ± SEM (n = 3, two-tailed Student's *t*-test, *P < 0.05, NS, not significant).

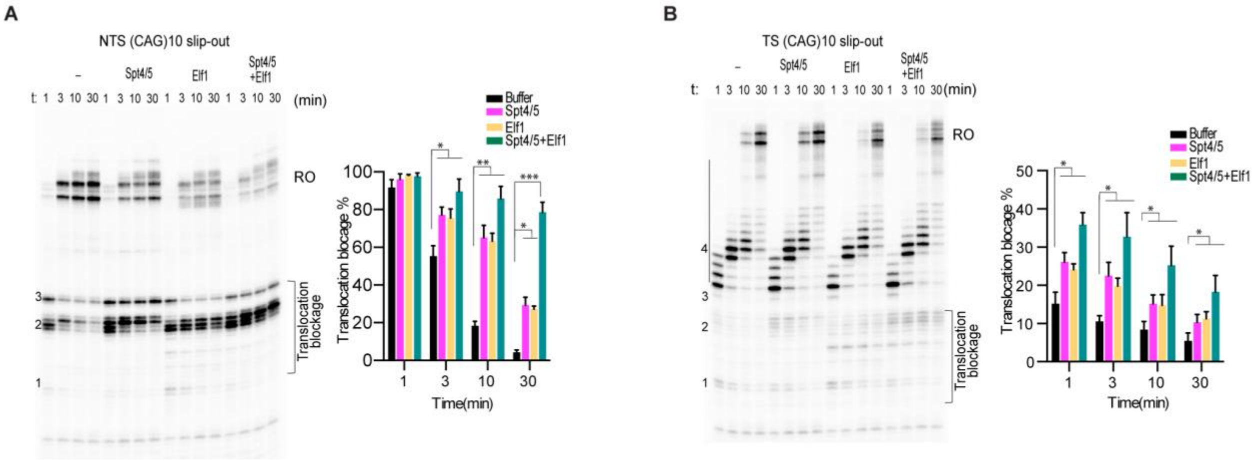

Figure S4. Spt4/5 and Elf1 inhibit transcription bypass of the $(CAG)_{10}$ slip-out structures. (A) Spt4/5 and Elf1 inhibit transcription bypass of NTS CAG slip-out individually and synergistically. (B) Spt4/5 and Elf1 inhibit transcription bypass of TS CAG individually and synergistically. Data are means ± SEM, n = 3. (n = 3, two-tailed Student's *t*-test, *P < 0.05, **P < 0.01).

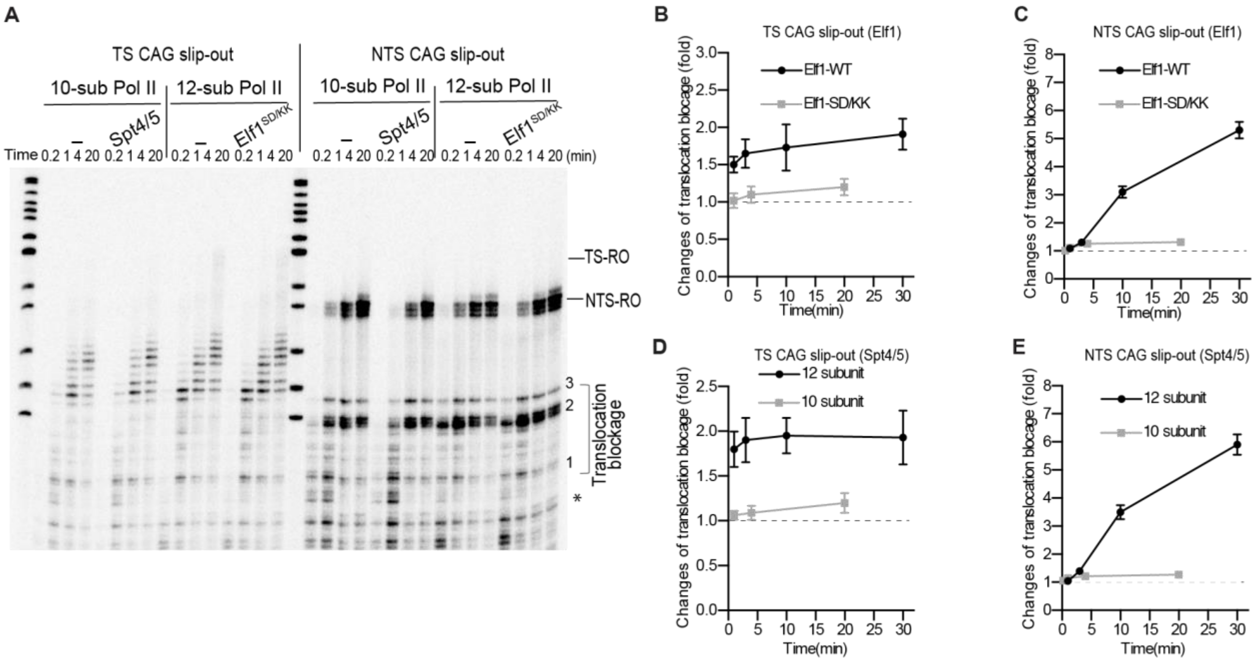

Figure S5. Inhibition of Pol II bypass of $(CAG)_{10}$ slip-out by Spt4/5 and Elf1 is dependent on the Spt4/5-Pol II interaction and Elf1-Pol II interaction. (*A*) Transcription assay with the protein mutants. $Elf1^{SD/KK}$ is an Elf1 mutant that is defective in Pol II interaction. Ten-subunit Pol II is a form of Pol II without Rpb4/7 (stalk). The stalk of Pol II is important for the interaction between Spt4/5 and Pol II. (*B-E*) Quantification of the transcription assay. (*B, C*) Comparison between the inhibitory effect of wild type and a mutant of Elf1 on 12-subunit Pol II bypass of the CAG slip-out structure. The transcriptional blockage efficiencies are normalized by transcriptional blockage efficiency in the absence of Elf1. The Elf1 mutant shows a dramatically reduced inhibitory effect. The result of wild type Elf1 is based on the results shown in Figure S2. (*D, E*) Comparison between the inhibitory effect of Spt4/5 on the 12-subunit Pol II and 10-subunit Pol II. The transcriptional blockage efficiencies are normalized by transcriptional blockage efficiency in the absence of Spt4/5 (12-subunit Pol II alone (black line) or 10-subunit Pol II alone (gray line), respectively). Spt4/5 shows a dramatically reduced inhibitory effect on 10-subunit Pol II than that of 12-subunit Pol II. The result of 12-subunit Pol II is based on the results shown in Figure S3.
